# Normal Reference Values of Tissue Doppler Imaging Parameters for Right Ventricular Function in Young Adults: a Population Based Study

**DOI:** 10.5812/cardiovascmed.9843

**Published:** 2013-10-28

**Authors:** Maryam Shojaeifard, Maryam Esmaeilzadeh, Majid Maleki, Hooman Bakhshandeh, Fatemeh Parvaresh, Nasim Naderi

**Affiliations:** 1 Echocardiography Research Center, Rajaie Cardiovascular Medical and Research Center, Iran University of Medical Sciences, Tehran, IR Iran; 2 Rajaie Cardiovascular Medical and Research Center, Iran University of Medical Sciences, Tehran, IR Iran; 3 Cardiac Electrophysiology Research Center, Rajaie Cardiovascular Medical and Research Center, Iran University of Medical Sciences, Tehran, IR Iran

**Keywords:** Right ventricle Function, Doppler Echocardiography, Tissue Doppler Imaging

## Abstract

**Background:**

Tissue Doppler imaging is used routinely to quantify both left and right ventricular function. However, normal reference values of echocardiography parameters of the right ventricle in Iranian population are still unknown.

**Objectives:**

Accordingly, we conducted a study to determine the normal values of echocardiography parameters of right ventricular function in a healthy Iranian population.

**Patients and Methods:**

One hundred and eighty seven healthy volunteer subjects enrolled. Normal subjects were chosen by taking into account history, physical examination, ECG and echocardiography.

**Results:**

Reference ranges (5th and 95th percentile values) for tricuspid E velocity, A velocity, E/A ratio, deceleration time, annular Sa velocity, Ea velocity, and E/Ea ratio were derived for the whole individuals and for each of the three age groups (< 30, 30–39, 40-49). The deceleration time, E/ Ea ratio and acceleration time of the iso-volumetric contraction time (IVA) were greater in male than in female. All measured parameters were bigger but not statistically significant in the 40-49 year-old group in comparison with the < 30 year-old group. Comparison of data between different groups showed no significant differences between the majority of data when they have been adjusted to body surface area, age and sex.

**Conclusions:**

The reference ranges presented for the echocardiography parameters of right ventricular function, albeit not conducted in a sizable sample of normal cases, will help to standardize the assessment of RV functions, particularly by tissue Doppler imaging.

## 1. Background

Importance of the right ventricular (RV) function has been partly underestimated. The main role of the RV is contribution into the normal cardiac pump function so as to maintain adequate pulmonary perfusion pressure and low systemic venous pressure in order to prevent tissue and organ congestion(1).The RV function may be impaired due to primary right-sided heart diseases or secondary to left-sided heart diseases (2) and is considered as a major determinant of the clinical outcome (3, 4).It is possible to assess the RV function invasively using high fidelity pressure catheters to measure the peak negative pressure/time change (dP/dt) and right sided filling pressures, but this method is invasive and load-dependent (5, 6). Echocardiography is now recognized as versatile diagnostic tool that enables us to quantify cardiac chamber size, ventricular mass, and function noninvasively in varying clinical settings. It has also become a virtual imaging technique to evaluate the right side of the heart. Guidelines for the Echocardiographic Assessment of the Right Heart in Adults were provided by the American Society of Echocardiography in conjunction with the European Association of Echocardiography and the Canadian Society of Echocardiography (7). However, most of these data are derived from American and European populations and because both physical and racial differences might have an inﬂuence on chamber size and function, it is important to evaluate the echocardiography parameters of specific populations. Reference values based on Asian populations have recently been reported (8), although there is no specific report for the Iranian population. 

## 2. Objectives

Accordingly, we designed and conducted a study, to determine the normal values for echocardiographic measurements of the right side of the heart. 

## 3. Patients and Methods

The present study evaluated 187 healthy volunteer subjects between 18 and 49 years of age. These healthy subjects were chosen by taking into account history, physical examination, ECG and comprehensive transthoracic echocardiography. The study was approved by the ethics committee of Rajaie Cardiovascular Medical and Research Center, and informed consent was obtained from all subjects.

### 3.1. Echocardiography study

All subjects were studied using M-mode and two-dimensional echocardiography and conventional Doppler and TDI in accordance with the American society of echocardiography guidelines (9).

**Tissue Doppler Imaging**: Myocardial velocities were measured on-line using a standard pulsewave Doppler echocardiography. Color-coded images were acquired during a breath hold over three consecutive cycles using tissue Doppler imaging at a frame rate of 150 MHz. The imaging angle was adjusted parallel with the myocardial segment of interest. The sample volume was placed at the junction of the RV free wall with the tricuspid annulus in the apical 4-chamber view. All the echocardiography measurements were performed using a General Electric Vivid 7 phased-array system (GE Medical Systems). Velocities were measured on-line at a sweep speed of 50-100 mm/s as if needed. All the parameters were calculated as the mean of three consecutive cycles. Following indices were determined by comprehensive Doppler inflow studies of both mitral and tricuspid valves: E wave deceleration time , E/A ratio , annular tissue Doppler velocities and the ratio of the early diastolic inflow velocity to the early tissue Doppler diastolic velocity (E/Ea).

The following measures of right ventricle were obtained additionally by TDI: iso-volumetric contraction time (IVCT); systolic annular velocity; early diastolic annular velocity; late diastolic annular velocity; and iso-volumetric relaxation time (IVRT). We measured different right-sided time intervals as following: acceleration time of the iso-volumetric contraction time (IVA); duration of S wave; time interval from the beginning of the QRS complex to the beginning of the early tissue Doppler diastolic velocity; duration of IVRT; time interval from the beginning of the IVCT to the beginning of the early tissue Doppler diastolic velocity. Mean TR velocity and IVC diameter and respiratory variation were measured to calculate pulmonary artery systolic pressure.

### 3.2. Statistical Analysis

Statistical analysis was performed using SPSS 15 for Windows (SPSS Inc. Chicago, Illinois), and the variables were expressed as mean ± SD. The Kolmogorov-Smirnov test was employed to check normal distribution of the continuous variables; standard statistical techniques was used to test the differences between patient subgroups with and without major cardiovascular events; Student t-test was used for the normally distributed continuous variables; and Mann-Whitney test for the non-normally distributed continuous and ordinal categorical variables. For all methods, a P value < 0.05 was considered statistically significant.

## 4. Results

### 4.1. Descriptive Data

Study subjects consisting of 136 (85%) female and 51 (15%) male. Tables 1  and 2 demonstrate the general characteristics and echocardiography data of the study population.The mean age was 32 ± 7.7 and 30 ± 6.9 years for female and male, respectively. Right heart PW Doppler and TDI measures (Mean ± SD) were as following: E wave = 57 ± 8 cm/s , A wave = 44 ± 8 cm/s, EDT = 209 ± 44 m/s, E/A ratio = 1.32 ± 0.2, E/ Ea = 4.6 ± 0.9, IVCT = 12 ± 0.6 cm/s, Sa velocity = 13.5 ± 0.2 cm/s, Ea velocity = 15.7 ± 0.15 cm/s, Aa velocity = 15 ± 0.14 cm/s, S wave duration = 276 ± 26 m/s, IVRT = 24 ± 15 m/s, time interval from the beginning of the IVCT to the beginning of Ea velocity = 346 ± 38 m/s, time interval from the beginning of the QRS wave to the beginning of Ea velocity = 397 ± 34 m/s, time interval from the beginning of the QRS wave to the beginning of the Evelocity = 404 ± 62 m/s, IVA = 34.5 ± 8 m/s, IVRT= 24 ± 15 m/s, IVC diameter = 1.4 ± 0.2 cm, systolic pulmonary artery pressure = 23.5 ± 2.5 mm Hg.

**Table 1.** General Characteristics of Subjects

Characteristics	Male (n =51)	Female (n = 136)
**Age, y**	30 ± 6.9 ^b^	32 ± 7.7
**Heart rate, bpm**	74 ± 4.4	77 ± 5.5
**BSA, m^2^**	1.88 ± 0.7	1.69 ± 0.65
**Systolic BP ^a^, mmHg**	119 ± 11	118 ± 8
**Diastolic BP, mmHg**	74 ± 8	71 ± 6
**LVEF ^a^, %**	59 ± 4	61 ± 4
**Mitral E/Ea ^a^**	6.5 ± 0.9	6.8 ± 0.9
**RVEDD ^a^, mm**	26.5 ± 3.9	27.3 ± 3
**TAPSE ^a^, mm**	25.9 ± 2.9	24.5 ± 3.2
**RV Sa ^a^, cm/s**	13.9 ± 0.6	14.2 ± 0.5
**TR ^a^ gradient, mmHg**	17 ± 3.2	16 ± 3.5

^a^ Abbreviations: BP ; blood pressure, LVEF; left ventricular ejection fraction, Mitral E/Ea; ratio of early mitral diastolic velocity to early diastolic mitral annular velocity, RVEDD; right ventricular end-diastolic diameter, TAPSE; tricuspid annular plane systolic excursion, Sa: tricuspid annular systolic velocity, TR; tricuspid regurgitation

^b^ Data are shown with Mean ± SD

**Table 2.** Echocardiographic Characteristics

Parameters	Mean ± SD	Percentile					Min	Max
		5	25	50	75	95		
**r E, cm/s ^a^**	0.57 ± 0.08	0.44	0.52	0.57	0.62	0.7	0.4	0.8
**r A, cm/s**	0.44 ± 0.08	0.32	0.37	0.43	0.5	0.59	0.23	0.65
**rEDT, m/s**	210 ± 42	176	188	200	210	308	126	418
**r E/A**	1.32 ± 0.2	1	1.2	1.3	1.4	1.7	0.93	1.84
**r E/Ea**	410 ± 35	351	382	414	437	460	306	522
**E/E, TV**	4.6 ± 0.9	1.6	4	4.6	5.2	6.4	0.6	7.2
**IVCT peak**	12 ± 0.6	8	10	12	13	18	5	18
**Sa peak, cm/s**	13.5 ± 0.2	10	12	13	15	17	9	20
**Ea peak, cm/s)**	15.7 ± 0.15	9	11	12	14	24	6	26
**Aa peak, cm/s**	15 ± 0.14	8	10	12	15	24	5	25
**IVA, cm/s**	34.5 ± 8	22	28	34	40	46	18	69
**S duration, m**	276 ± 26	237	262	274	291	319	192	363
**r IVRT, m/s**	24 ± 15	0	15	23	31	61	0	80
**r Q –Ea, m/s**	397 ± 34	342	374	400	416	462	320	493
**IVCT-Ea, m/s**	346 **± **38	290	322	345	369	413	203	466
**IVC, cm**	1.4 **±** 0.2	1.2	1.3	1.4	1.57	1.74	0.8	2
**PAP, mmHg**	23.5 **±** 2.5	20	20	25	25	25	15	30

^a^Abbreviations: r E ; tricuspid early diastolic velocity, r A; tricuspid late diastolic velocity, rEDT; tricuspid early diastolic deceleration time, r E/A ; the ratio of early to late tricuspid diastolic velocity, r E/Ea; ratio of early tricuspid diastolic velocity to early diastolic tricuspid annular velocity, Sa peak; tricuspid annular systolic velocity; Ea peak; tricuspid annular early diastolic velocity, Aa peak;tricuspid annular late diastolic velocity, IVA; the acceleration time of isovolumetric contraction, S duration; duration of tricuspid annular systolic velocity, r IVRT; right ventricular isovolumetric relaxation time; r Q –Ea :time interval form the beginning of QRS complex to the beginning of early tricuspid annular diastolic velocity; IVCT-Ea, time interval from the beginning of the isovolumetric relaxation time to the beginning of early tricuspid annular diastolic velocity, IVC; inferior vena cava, PAP; pulmonary artery pressure.

### 4.2. Sex Differences

There were statistically significant differences between male and female regarding *r *EDT, *r *E/ Ea , IVA, time interval from the beginning of the QRS complex to the beginning of Ea velocity, and IVC dimension (Table 3). The deceleration time, E/ Ea, IVA, and IVC dimension were greater in male than in female, but the time interval from the beginning of the QRS complex to the beginning of Ea velocity was smaller in men than those of women (Table 3). The remaining measures did not significantly differ between the two sexes.

**Table 3.** Echocardiographic Characteristics Based on Gender

Parameters	Female (n = 136)	Male (n = 51)	P value
**MV E/Ea ** **^a^**	6.8 ± 0.9 ^b^	6.5 ± 0.9	0.9
**r E ^a^, cm/s**	0.57± 0.07	0.55 ± 0.07	0.7
**r A ^a^, cm/s**	0.44 ± 0.08	0.43 ± 0.09	0.5
**IVCT-Ea, m/s**	194 ± 9	196 ± 11	0.002
**r E/A^a^**	1.3 ±0.15	1.35 ± 0.25	0.2
**r Q –E ^a^ , m/s**	408 ± 5	380 ± 5	0.4
**r E/Ea**	4.6 ± 0.7	4.8 ± 1	0.01
**IVCT peak, cm/s**	11.5 ± 2.9	11.6 ± 2.9	0.12
**Sa ^a^ peak, cm/s**	13.3 ± 2	13.4 ± 2	0.5
**Ea ^a^ peak, cm/s**	12.6 ± 2	11.8± 2	0.7
**Aa ^a^ peak, cm/s**	12.3 ± 1	11.2 ± 1	0.9
**IVA ^a^, cm/s**	32 ± 6	35 ± 7	0.03
**S duration ^a^ , m/s**	275 ± 22	271 ± 17	0.7
**r IVRT ^a^, m/s**	21 ± 10	19 ± 10	0.3
**r Q –Ea ^a^, m/s**	398 ± 32	380 ± 31	0.01
**IVCT-Ea ^a^, m/s**	347 ± 33	331 ± 35	0.6
**IVC ^a^, cm**	1.48 ± 0.14	1.57 ±0.18	< 0.001
**PAP ^a^, mmHg**	23.8 ±4.5	23.7 ± 4.8	0.8

^a^ Abbreviation: MV E/Ea ; the ratio of mitral early diastolic inflow velocity to mitral annular early diastolic velocity, r E ; tricuspid early diastolic velocity, r A; tricuspid late diastolic velocity, rEDT; tricuspid early diastolic deceleration time, r E/A ; the ratio of early to late tricuspid diastolic velocity, r Q-E; time interval between Q wave of ECG recording and tricuspid early diastolic velocity, r E/Ea; ratio of early tricuspid diastolic velocity to early diastolic tricuspid annular velocity, Sa peak; tricuspid annular systolic velocity; r Q –E; time interval form the beginning of QRS complex to the beginning of early mitral annular diastolic velocity, Ea peak; tricuspid annular early diastolic velocity, Aa peak;tricuspid annular late diastolic velocity, IVA; the acceleration time of isovolumetric contraction, S duration; duration of tricuspid annular systolic velocity, r IVRT; right ventricular isovolumetric relaxation time; r Q –Ea :time interval form the beginning of QRS complex to the beginning of early tricuspid annular diastolic velocity; IVCT-Ea, time interval from the beginning of the isovolumetric relaxation time to the beginning of early tricuspid annular diastolic velocity, IVC; inferior vena cava, PAP;pulmonary artery pressure.

^b^ Data are shown in Mean ± SD

### 4.3. Relation with Age 

Three age groups were defined (<30, 30–39, 40-50). Table 4 depicts differences in measures between the groups. In evaluation of the echocardiography data of the age groups, there was a statistically significant relationship only between the duration of Sa and age. All measured parameters were much bigger statistically in the 40-49 year-old group in comparison with the < 30 year-old group. No statistically significant relationship was detected between the other aforementioned echocardiography parameters and age (Table 4).

**Table 4.** Echocardiographic Characteristics Based on Age

Parameters	Age, y			P value
	18 - 29	30 - 39	40 - 49	
**MV E/Ea ** ^a^	6.4 ± 0.8 ^b^	6.7± 0.9	7.3 ± 0.8	0.001
**r E ^a^, cm/s**	0.58 ± 0.07	0.56 ± 0.08	0.56 ± 0.07	0.24
**r A ^a^, cm/s**	0.45 ± 0.09	0.43 ± 0.08	0.44 ± 0.07	0.35
**rEDT ^a^, m/s**	191 ± 26	197 ± 11	197 ± 14	0.3
**r E/A ^a^**	1.3± 0.19	1.3 ± 0.19	1.28 ± 0.17	0.53
**r Q –E ^a^, m/s**	391 ± 78	413 ± 31	407 ± 76	0.13
**r E/Ea ^a^**	4.5 ± 1	4.6 ± 0.9	4.9 ± 0.7	0.6
**IVCT ^a^ peakcm/s**	11 ± 2.6	12 ± 3.3	12 ± 2.7	0.5
**Sa ^a^ peak, cm/s**	13.7 ± 2.2	13.6 ± 1.7	12.5 ± 1.7	0.7
**Ea ^a^ peak, cm/s**	13 ± 2.5	12 ± 2	11 ± 1.9	0.25
**Aa ^a^ peak, cm/s**	11.2 ± 3.5	11.9 ± 3	14 ± 4	0.5
**IVA ^a^ , cm/s**	31.7 ± 7	33.7 ± 5.8	33.2 ± 6.4	0.3
**S duration ^a^, m/s**	272 ± 19	272 ± 22	281 ± 23	0.005
**r IVRT ^a[Table-fn fn4977]^, m/s**	20 ± 13	21.4 ± 9.3	21.6 ± 8.5	0.6
**r Q –Ea ^a^, m/s**	392 ± 36	395 ± 27	395 ± 35	0.95
**IVCT-Ea ^a^, m/s**	344 ± 34	341 ± 27	346± 41	0.7
**IVC ^a^, cm**	1.5 ± 0.14	1.5 ± 0.16	1.53 ± 0.17	0.4
**PAP ^a^, mmHg**	23.4 ± 2.3	23.8 ± 2.1	24.4 ± 1.6	0.2

^a^ Abbreviation: MV E/Ea ; the ratio of mitral early diastolic inflow veloity to mitral annular early diastolic velocity, r E ; tricuspid early diastolic velocity, r A; tricuspid late diastolic velocity, rEDT; tricuspid early diastolic deceleration time, r E/A ; the ratio of early to late tricuspid diastolic velocity, r Q-E; time interval between Q wave of ECG recording and tricuspid early diastolic velocity, r E/Ea; ratio of early tricuspid diastolic velocity to early diastolic tricuspid annular velocity, Sa peak; tricuspid annular systolic velocity; r Q –E; time interval form the beginning of QRS complex to the beginning of early mitral annular diastolic velocity, Ea peak; tricuspid annular early diastolic velocity, Aa peak;tricuspid annular late diastolic velocity, IVA; the acceleration time of isovolumetric contraction, S duration; duration of tricuspid annular systolic velocity, r IVRT; right ventricular isovolumetric relaxation time; r Q –Ea :time interval form the beginning of QRS complex to the beginning of early tricuspid annular diastolic velocity; IVCT-Ea, time interval from the beginning of the isovolumetric relaxation time to the beginning of early tricuspid annular diastolic velocity, IVC; inferior vena cava, PAP;pulmonary artery pressure

^b^ Data are shown in Mean ± SD

### 4.4. Relation with the Body Surface Area 

All measurements were classified based on body surface area in the three groups (Table 5). Comparison of data between different groups after adjustment to body surface area showed no significant differences between the majorities of data. 

**Table 5.** Echocardiographic Characteristics Based on Body Surface Area

Parameters	Body Surface Area			P value
	<1.5 m ^2^	1.5 – 1.9 m ^2^	≥ 2 m ^2^	
**MV E/Ea** ^a^	6.1 ± 0.8 ^b^	6.8 ± 0.9	6.74 ± 1	0.4
**r E ^a^, cm/s**	0.54 ± 0.06	0.57±0.07	0.56 ± 0.08	0.4
**r A ^a^, cm/s**	0.42 ± 0.06	0.44 ± 0.08	0.46 ± 0.08	0.25
**rEDT ^a^, m/s**	192 ± 11	194 ± 20	200 ± 11	0.56
**r E/A ^a^**	1.3 ± 0.11	1.32 ± 0.2	1.23 ± 0.15	0.3
**r Q –E ^a^, m/s**	406 ± 31	404 ± 61	379± 10	0.4
**r E/Ea ^a^**	4.38 ±1	4.68 ± 0.9	4.64 ± 1.1	0.4
**IVCT peak, cm/s**	10 ± 1.2	11.5 ± 3	12 ± 3	0.014
**Sa ^a^ peak , cm/s**	13 ± 1.3	13.3 ± 2	13.7 ± 2	0.7
**Ea ^a^ peak, cm/s**	12.6 ± 2.1	12.4 ± 2.3	12.4 ± 2.4	0.56
**Aa ^a^ peak, cm/s**	11 ± 3.3	12.2 ± 3.8	12 ± 2.5	0.98
**IVA ^a^ , cm/s**	33 ± 8	32 ± 6.2	34 ± 8.4	0.32
**S duration ^a^, m/s**	270 ± 16	275 ± 21	267 ± 17	0.04
**r IVRT ^a^, m/s**	182 ± 9.7	21.6 ± 11	16 ± 10	>0.99
**r Q–Ea ^a^,m/s**	390 ± 33	396± 33	375 ± 29	0.4
**IVCT-Ea ^a^,m/s**	347 ± 37	346 ± 33	317 ± 26	0.1
**IVC ^a^, cm**	1.4 ± 0.13	1.5 ± 0.15	1.5 ± 0.17	0.8
**PAP ^a^, mmHg**	24.3 ± 1.7	23.7 ± 2.2	24.6 ± 1.3	0.4

^a^ Abbreviations: MV E/Ea ; the ratio of mitral early diastolic inflow velocity to mitral annular early diastolic velocity, r E ; tricuspid early diastolic velocity, r A; tricuspid late diastolic velocity, rEDT; tricuspid early diastolic deceleration time, r E/A ; the ratio of early to late tricuspid diastolic velocity, r Q-E; time interval between Q wave of ECG recording and tricuspid early diastolic velocity, r E/Ea; ratio of early tricuspid diastolic velocity to early diastolic tricuspid annular velocity, Sa peak; tricuspid annular systolic velocity; r Q –E; time interval form the beginning of QRS complex to the beginning of early mitral annular diastolic velocity, Ea peak; tricuspid annular early diastolic velocity, Aa peak;tricuspid annular late diastolic velocity, IVA; the acceleration time of isovolumetric contraction, S duration; duration of tricuspid annular systolic velocity, r IVRT; right ventricular isovolumetric relaxation time; r Q –Ea :time interval form the beginning of QRS complex to the beginning of early tricuspid annular diastolic velocity; IVCT-Ea, time interval from the beginning of the isovolumetric relaxation time to the beginning of early tricuspid annular diastolic velocity, IVC; inferior vena cava, PAP; pulmonary artery pressure

^b^ Data are shown in Mean ± SD

## 5. Discussion

Diastole is the period of ventricular relaxation and encompasses periods of iso-volumetric relaxation and early and late diastolic filling. Impaired diastolic function with relative preservation of the systolic function is an early feature of either the right and or left ventricular impairment (10). The RV dysfunction has been shown to predict decreased survival in patients with congestive heart failure. Most studies conducted thus far, have focused primarily on the RV systolic dysfunction (11).Right ventricular diastolic dysfunction has been found to occur in patients with chronic pulmonary disease and pulmonary thrombo embolism, systolic left ventricular failure, and systemic sclerosis (10). With the introduction of Doppler echocardiography, other parameters besides clinical assessment, estimation of RA pressure regarding IVC diameter and collapsibility have been examined as measures of RV performance. Previous studies had grate tendency to evaluate RV diastolic function conventionally using trans-tricuspid Doppler flow velocity profile. The variables measured include the peak velocity of early filling (E), peak velocity of late filling due to atrial contraction (A), E/A ratio, and deceleration time of early filling (EDT). Since the velocities across the tricuspid valve are significantly lower than those across the mitral valve, hence rEDT is longer than the mitral EDT. The tricuspid flow parameters do not appear to be affected by age (7) but respiration causes pronounced variability, so all measurement should be made at end expiration. Up to present time only a few studies have been carried out on RV function in normal subjects. No investigation was done in our country in order to achieve normal reference values of RV function on a substantial number of normal young adult individuals. This study demonstrated changes in Pulsed Wave and tissue Doppler parameters with age, sex and body surface area in a single healthy population. Gender differences and age-related changes in echocardiography parameters have been evaluated in our study. Our values were closely resembled to values of guidelines for the echocardiographic assessment of the right heart endorsed by the European association of echocardiography (7). The mean value of peak E and rA velocity in our study were similar to those of which mentioned in the European guidelines (7) [57 ± 8 cm/s versus 54 (52-56) cm/s and 44 ± 8 cm/s versus 40 (38-41) cm/s respectively]. In our study mean r E/A ratio was nearly close to European guidelines [1.32 ± 0.2 versus 1.4 (1.4-1.5)] but rEDT was rather longer than that those of Europeans (209± 44 m/s versus 174 (163-186) m/s) which seems to be mostly secondary to the considerable difference between mean age of study populations (31 ± 7.3 versus 58 ± 19 years for PW Doppler and 64.3 ± 9.5 for color TDI measures in European studies). On the other hand Ea , Aa and r E/ Ea ratio showed no significant difference compared with the European guideline (15.4 cm/s versus 14 (13-14) , 15.7 ± 0.15 cm/s versus 13 (12-14) and 4.6 ± 0.9 versus 4 (4-4) respectively). Also comparison of data between different groups showed no significant differences between the majority of data when they have been adjusted to body surface area, age and sex. There are only a few limitations for present study. Firstly the number of subjects was not as enough as for a cohort study. Secondly older subjects were not enrolled into the study, so future study in normal subjects more than fifty-years old should be conducted to obtain the normal reference values in old healthy adults to assess how aging affects these parameters. We herein report for the ﬁrst time, the normal reference values of echocardiography parameters of right ventricle in normal young adults in Iranian population. The reference ranges presented for the echocardiography parameters of right ventricular function, albeit not conducted in a sizable population, will help to standardize the assessment of RV function, particularly by tissue Doppler imaging not only in routine echocardiography examinations, but also in interpreting the results of clinical trials.
